# Foot Drop, Wrist Drop, and Digital Ischemia As Initial Manifestations in Sjögren’s Disease

**DOI:** 10.7759/cureus.79641

**Published:** 2025-02-25

**Authors:** Rafeef R Alsheikhmubarak, Asmaa Hegazy, Fahad Aleidan, Saitah F Alshammari

**Affiliations:** 1 Internal Medicine, King Saud University, Riyadh, SAU; 2 Internal Medicine, Rheumatology, and Immunology, King Saud Medical City, Riyadh, SAU; 3 Medicine, Prince Sultan Military Medical City, Riyadh, SAU

**Keywords:** foot drop, polyneuropathy, rheumatology & autoimmune diseases, sjögren’s syndrome, wrist drop

## Abstract

A 50-year-old Saudi woman, with a history of rheumatoid arthritis (RA), presented to the emergency room with a three-week history of progressive weakness. Her symptoms began with difficulty elevating her arms and standing up from a chair. Over the next two days, her weakness progressed, leading to bilateral wrist drop and foot drop, along with an inability to raise her hands or stand. She also reported sensory changes, including decreased sensation to touch and heat, most prominent in the lower limbs, and complaints of dry eyes and mouth. Additionally, the patient noted bluish discoloration of her right middle finger. Physical examination revealed bilateral wrist and foot drop, decreased distal strength, absent reflexes, and a black discoloration on the distal interphalangeal (DIP) joint of her right middle finger.

The patient had a history of inflammatory polyarthritis diagnosed as RA two years prior. Her physical findings, along with dry eyes, mouth, and the characteristic sensory and motor deficits, raised concern for Sjögren’s syndrome (SS). Diagnostic tests confirmed SS, showing positive salivary gland biopsy results with lymphocytic infiltration, a positive Schirmer test for dry eyes, and positive serum autoantibodies for anti-Ro, anti-La, and ANA. Nerve conduction studies revealed severe motor and sensory polyneuropathy.

Treatment included a three-day pulse of steroids, followed by oral prednisone, and cyclophosphamide for six months. Additionally, the patient was treated for osteoporosis with teriparatide, then switched to denosumab, and managed with methotrexate and supportive therapies, including artificial tears and physiotherapy. The patient showed significant improvement in symptoms and responded excellently to the treatment.

## Introduction

Sjögren’s syndrome (SS) is a chronic autoimmune disorder primarily characterized by immune-mediated destruction of the exocrine glands, leading to hallmark symptoms of xerophthalmia (dry eyes) and xerostomia (dry mouth). However, beyond its classic glandular involvement, SS is increasingly recognized as a systemic disease with the potential to affect multiple organ systems, including the musculoskeletal, pulmonary, renal, and nervous systems. Neurological manifestations, while less common, can significantly impact disease progression and patient quality of life [1,2]. During the literature review, only a few cases were found to have neurological manifestations as presenting symptoms.

In this case report, we present a 50-year-old female with a known history of rheumatoid arthritis (RA) who was admitted to the emergency room with predominant neurological symptoms, including weakness, wrist drop, and foot drop. Given the rarity of such a presentation in SS and its potential to be misdiagnosed or overlooked, this case contributes to the growing body of evidence emphasizing the diverse and heterogeneous clinical spectrum of SS. By discussing the diagnostic challenges and relevant literature, we aim to highlight the importance of considering SS in the differential diagnosis of unexplained peripheral neuropathy, particularly in patients with a history of autoimmune disease. 

## Case presentation

A 50-year-old married Saudi housewife presented to the ER with a three-week history of progressive weakness. The problem started with a decreased ability to elevate her hands to comb her hair and difficulty in getting up from the chair. Two days prior to the presentation, the weakness worsened, and she was unable to raise her hands upward, which remained in a hanging position with a bilateral wrist drop. Additionally, she was unable to stand on her feet due to bilateral foot drop and was unable to move them upward.

Additionally, she reported decreased sensation to touch and heat in all four limbs, more pronounced in the lower limbs, as well as dry eyes and mouth. She also noted bluish discoloration of her right middle finger, which was first noticed two weeks ago and was not painful. Moreover, the patient developed inflammatory polyarthritis two years ago and was diagnosed with RA at that time. Apart from that, she had no history of oral ulcers, patchy hair loss, Raynaud phenomenon, photosensitivity, malar rash, deep vein thrombosis (DVT), cerebrovascular accident (CVA), or abortions.

**Figure 1:**
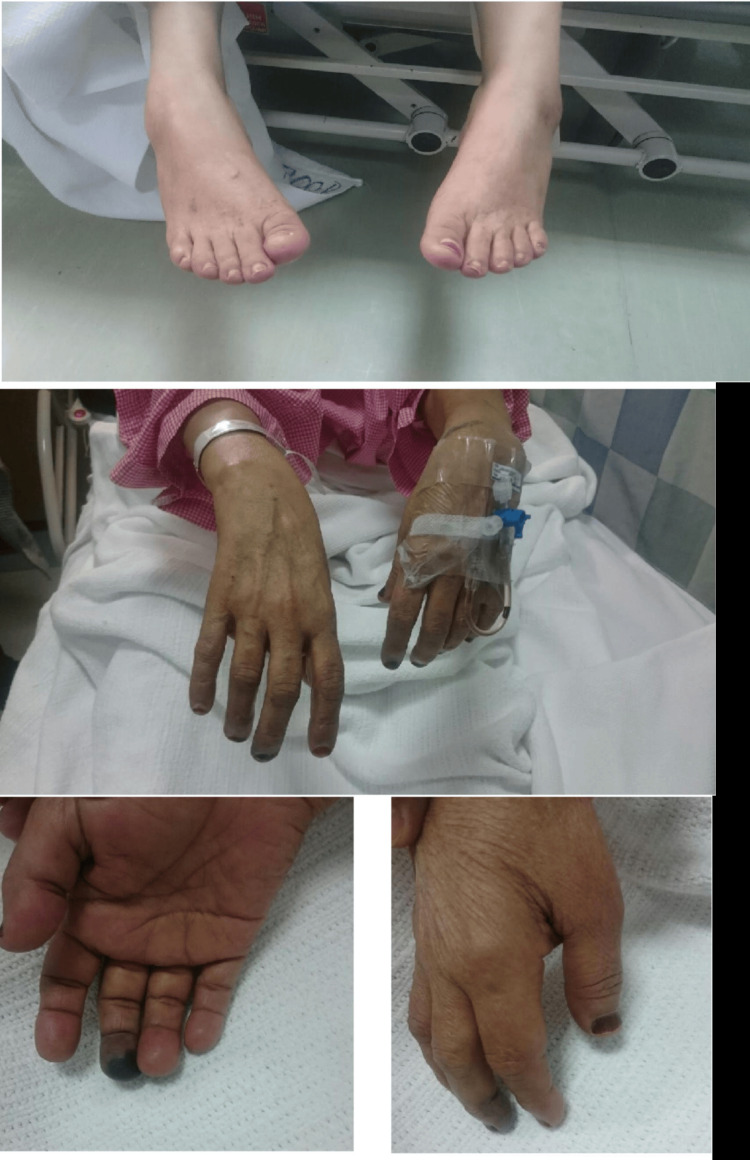
Bilateral foot drop, bilateral wrist drop, and digital ischemia

Upon physical examination, the patient was alert, oriented, well-nourished, and in no acute distress. Vital signs were stable. The skin was warm with no pallor, cyanosis, or jaundice. The patient showed bilateral foot and wrist drops (Figure 1). In addition, she had decreased power, more pronounced distally, and a global decrease in reflexes with a mute plantar reflex. She had decreased sensation to touch and temperature in a glove-and-stocking distribution. She also had black discoloration reaching the distal interphalangeal (DIP) joint of her right middle finger (Figure 1). The systematic examination was otherwise unremarkable.

Laboratory investigations were performed, with the results detailed in Table 1.

**Table 1:** Laboratory investigation results

Test	Result	Normal level
Hemoglobin, g/dL	13.5	12-16
White blood cells, L^-1^	7x10^9^	4.5 to 11.0
Platelet, L^-1^	401x10^9^	150-450
Prothrombin time (PT), seconds	15.3	11-13
Partial thromboplastin time (PTT), seconds	37.4	25-35
International normalized ratio (INR), seconds	1.16	<1
Urea, mmol	6	1.8-7.1
Creatinine, µmol/L	74	53-97.2
Sodium, mEq/L	133	135-145
Potassium, mEq/L	3.8	3.5-5.2
Albumin, mg/dL	23	11-48
24-hour urine protein, mg\day	768	<150
Aspartate aminotransferase (AST), U\L	64	10-36
Alkaline phosphatase (ALP), U\L	258	44-147
Alanine aminotransferase (ALT), U\L	46	7-56
Antinuclear antibody (ANA)	1/1280	Negative
Rheumatoid factor (RF)	35	Negative
Anti-cyclic citrullinated peptide (CCP)	Negative	Negative
Anti-Ds-DNA	Negative	Negative
Perinuclear anti-neutrophil cytoplasmic antibody (pANCA)	Negative	Negative
Cytoplasmic anti-neutrophil cytoplasmic antibody (cANCA)	Negative	Negative
Lupus anticoagulant	Negative	Negative
Anti-smith	Negative	Negative
Anti-JO-1	Negative	Negative
Anti-ribonucleoprotein (RNP)	Negative	Negative
Anti-mitochondrial antibody (AMA)	Negative	Negative
Anti-smooth muscle antibody (ASMA)	Negative	Negative
Anti-Sjögren’s syndrome-related antigen A (SSA)	149	Negative
Anti-Sjögren’s syndrome-related antigen B (SSB)	137	Negative
C-reactive protein (CRP), mg/L	44.6	8-10
Erythrocyte sedimentation rate (ESR), mm\hr	70	<30
IgG, mg\dL	983	600-1600
C3	Normal	
C4	Low	

The patient was admitted and underwent a nerve conduction study (NCS), which showed severe motor and sensory polyneuropathy with no action potential. Her minor salivary gland (MSG) biopsy results showed 10 lymphocytic aggregates, each containing more than 50 lymphocytes. The Schirmer test was positive for the right eye (0/5) and left eye (1/5). Ultrasound of the hands showed synovitis of the wrist joints but no erosions and the DEXA scan revealed osteoporosis.

The diagnostic criteria for SS include the presence of serum autoantibodies and lymphocytic sialadenitis on labial salivary gland biopsy. Our patient met both criteria, with a positive biopsy and positive serum antibodies for anti-Ro (Sjögren’s syndrome-related antigen A (SSA)) and anti-La (Sjögren’s syndrome-related antigen B (SSB)), which are highly specific for SS and often found together, further supporting the diagnosis. Additionally, she tested positive for antinuclear antibody (ANA) and rheumatoid factor (RF), with low C4 and normal C3 (Table 1).

The patient received a steroid pulse for three days, followed by oral prednisone at 1 mg/kg. Cyclophosphamide was administered at 1 g for six months. For osteoporosis, the patient was prescribed teriparatide for 18 months, after which she was switched to denosumab every six months. Maintenance medications included methotrexate, folic acid, vitamin D, calcium carbonate, omeprazole, artificial tears, and physiotherapy. The patient responded excellently to the treatment.

## Discussion

The pathophysiology of SS is not yet fully understood, but a complex interaction between susceptible individuals and environmental factors plays a significant role [1]. It is well established that SS can have a wide range of signs and symptoms, as it is a multisystemic disease [2]. Nearly 71% of SS patients will develop extraglandular manifestations [2].

A cohort study conducted in France aimed to estimate the prevalence of neurological manifestations among SS patients. The study included 392 patients diagnosed with SS, and the results showed that 16.1% had peripheral nervous system (PNS) involvement, and 3.6% had central nervous system (CNS) involvement [3]. In a systematic review, the prevalence of peripheral neuropathies among patients with SS was found to be 15%, with 83% of those affected being females. The most common type of peripheral neuropathy was distal axonal neuropathy, with a prevalence of 80%, followed by sensory ganglionopathy [4].

In a study conducted in Germany, 184 patients with polyneuropathies were investigated for SS, and 44 were diagnosed. Among those diagnosed, weakness was reported in only 11 (25%) cases [5].

Few case studies have reported weakness in association with SS [6,7]. However, in this case report, the patient experienced weakness along with bilateral foot and wrist drops.

While some cases have been reported of patients presenting with bilateral wrist drops, these instances have been attributed to other conditions. For example, one case reported a patient who presented with bilateral wrist drop, pain, and paresthesia in both hands. The patient was later diagnosed with Behçet's disease, which responded well to treatment with cyclophosphamide [8]. In another case, a patient with bilateral foot and wrist drops, weakness, and numbness was diagnosed with severe sensorimotor polyneuropathy secondary to SLE-associated vasculitis [9]. To conclude, as far as we know, there are no documented cases in the literature associating wrist or foot drops with SS.

## Conclusions

This case report highlights a rare presentation of SS with bilateral wrist and foot drop, progressive weakness, and sensory disturbances. While peripheral neuropathy is a recognized complication of SS, such a presentation is rarely documented. This case underscores the importance of considering neurological complications in SS to ensure timely diagnosis and management. Furthermore, it contributes to the existing literature by broadening the spectrum of possible neurological manifestations. Recognition of such cases is crucial, as early diagnosis and appropriate immunosuppressive treatment can significantly impact patient outcomes.
